# Eccrine carcinomas and homologous recombination repair cancer predisposition genes: A case series

**DOI:** 10.1016/j.jdcr.2025.04.047

**Published:** 2025-07-12

**Authors:** Renée Morecroft, Jordan Phillipps, Kaiden Barozinsky, Susan Jones, Lulu Sun, Danielle Crites, Omar Butt, George Ansstas, Patrick Grierson, Tanner M. Johanns, Alice Zhou

**Affiliations:** a Department of Internal Medicine, HCA Florida Orange Park Hospital, Orange Park, Florida; b Department of Dermatology, Mayo Clinic, Jacksonville, Florida; c Division of Medical Oncology, Washington University in Saint Louis, St. Louis, Missouri; d Department of Pathology and Immunology, Washington University in Saint Louis, St. Louis, Missouri

**Keywords:** cancer predisposition genes, eccrine carcinoma, homologous recombination repair, PARP inhibitor, screening

## Introduction

Eccrine carcinomas (ECs) account for less than 0.01% of cutaneous malignancies, with little understanding of their malignant transformation and genetic landscape.^1^ While most ECs, like other cutaneous adnexal carcinomas, exhibit localized disease, they can progress to metastatic disease. The rate at which these tumors metastasize varies depending on the subtype and other factors.^2^ According to the literature, eccrine porocarcinoma has a metastatic rate to regional lymph nodes of approximately 20% and a distant metastasis rate of about 10%.^2^ Another study reported that metastatic disease at presentation is not uncommon, with a rate of 22%.^2^ Metastatic EC (mEC) has 60% to 70% mortality and responds poorly to existing therapies.^1^

This case series analyzed molecular genotyping in 2 patients with mEC at initial presentation, with partner and localizer of breast cancer gene 2 (*PALB2)*, breast cancer gene 1 (*BRCA1*), and postmeiotic segregation increased 2 (*PMS2)* germline mutations—homologous recombination deficiency (HRD) genes.^3^ These mutations are linked to cancer predisposition syndromes, underscoring the need for genetic counseling for patients and their families. Given the effectiveness of poly(ADP-ribose) polymerase inhibitors (PARPi) in HRD-related cancers such as *BRCA1*/*BRCA2*-mutated breast and ovarian cancers, this study suggests PARPi as a potential treatment for EC.^4^ It highlights the importance of molecular genotyping in understanding and managing this rare malignancy.

## Case series

We identified 2 patients at the Siteman Cancer Center, Washington University School of Medicine, who presented for mEC management. Both had HRD-associated cancer predisposition genes, with 1 responding to PARPi. Treatments were well-tolerated. Patient demographics and clinical data are reported in Table I.

**Table I:** Patient demographics and clinical data

Patient demographics		
Cases	**Case 1**	**Case 2**
Age, gender, and race	58/Male/Caucasian (non-Hispanic)	88/Male/Asian
Prior cancer history	Renal cell carcinoma treated with definitive nephrectomy 3 y prior to presentation	None
Family history	2 first female cousins on either side of his family with breast cancer	None
Diagnosis	Stage 4 eccrine carcinoma	Stage 4 eccrine carcinoma (hidradenocarcinoma/porocarcinoma)
Therapies and response	1. Radiation therapy to the right humeral head and completed 6 cycles of docetaxel, followed by maintenance enzalutamide. Response: disease progression Complications: none 2. Combination ipilimumab/nivolumab therapy Drug class: ICI Response: none AE: immunotherapy-associated adrenal insufficiency and hypothyroidism.	1. Olaparib, switched to talazoparib Drug class: PARPi Response: partial response

*AE*, Adverse effects; *ICI*, immune checkpoint inhibitor; *PARPi*, poly (ADP-ribose) polymerase inhibitor.

### Case 1

**Fig 1:**
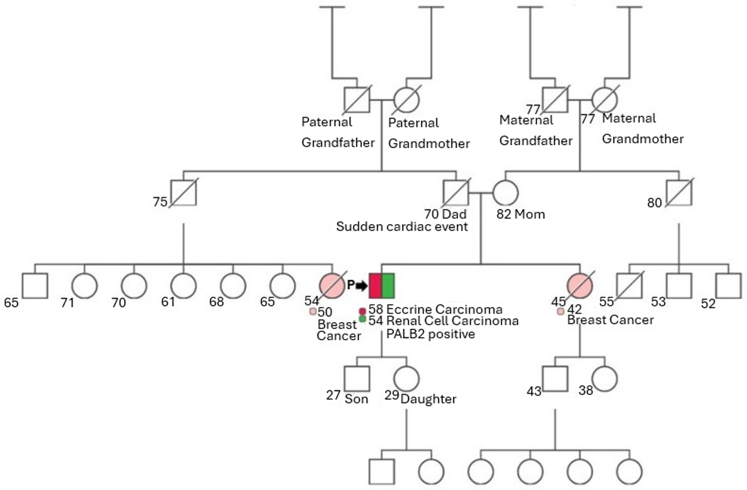
Pedigree chart showing the familial oncologic history of case 1. *P*, Patient.

A 58-year-old male with prior stage I papillary renal cell carcinoma (RCC), treated with nephrectomy, presented with a cutaneous axillary and single bony lesion. Bone biopsy revealed metastatic adenocarcinoma (cytokeratin [CK] 7-positive; paired box gene [PAX] 8-, RCC antibody-, CK20-, thyroid transcription factor-1 [TTF1]-negative). The axillary lesion, an ulcerated invasive adenocarcinoma, had appeared 6 months earlier. Positron emission tomography imaging showed a 1.4 cm 2-fluoro-2-deoxy-d-glucose-avid left axillary lymph node. Initially suspected to have metastatic dedifferentiated RCC, he was treated with ipilimumab/nivolumab but progressed, developing immune-related adrenal insufficiency and hypothyroidism. He was then referred to a tertiary cancer center. Left axillary resection with lymph node dissection confirmed primary EC (CK7-positive and PAX8-, RCC-, CK20-, TTF1-, estrogen receptor-, and progesterone receptor-negative). Histopathology of the humeral lesion resembled the left axillary lesion but differed from RCC (Table II), confirming mEC. Postresection, he received radiation to the right humeral head. Restaging showed progression, prompting 6 cycles of docetaxel, with an elicited short-term response. Further testing on the axillary tumor showed androgen receptor overexpression (90%) and human epidermal growth factor 2 (HER2) negativity. Next-generation sequencing (Caris) identified pathogenic *PALB2* (p.Met723fs and p.Val504fs) and phosphoinositide-3-kinase (*PIK3CA)* (p.His1047Arg) variants and a *BRCA1 p.*Thr528Pro variant of uncertain significance (VUS). Immunohistochemistry (IHC) showed intact mismatch repair proteins and low tumor mutational burden (7mut/Mb). Germline testing (Tempus xG+) confirmed a pathogenic heterozygous *PALB2* (p.Met723Valfs) mutation, consistent with *PALB2*-related cancer susceptibility (Table II). This germline mutation was present in his RCC but without loss of heterozygosity, suggesting it was not *PALB2-*driven. A limited family history revealed breast cancer in a sister and a paternal female first cousin (Fig 1). Following docetaxel, he received enzalutamide maintenance due to androgen receptor overexpression. Restaging revealed 2-fluoro-2-deoxy-d-glucose-avid hepatic and pancreatic lesions, confirming metastatic pancreatic acinar carcinoma. Treatment priority shifted to his pancreatic cancer, and he was started on modified fluorouracil/leucovorin/irinotecan (150 mg/m^2^)/oxaliplatin every 14 days given his *PALB2* mutation history.^5^ At 6 months, restaging showed stable pancreatic disease, though his EC had bony progression. This was managed with radiation therapy. Future treatment options for both pancreatic carcinoma and EC, upon progression, would include PARP inhibition.

**Table II:** Specimen histology and pathology details

Specimen site	IHC	Histology	Diagnosis	Mutations
Case 1				
Nephrectomy	Positive for CK7, P504S Negative for CA-IX and c-KIT		Papillary renal cell carcinoma	
Right humerus	Positive: CK 7 Negative: PAX 8, RCC antibody, CK20, TTF1		Metastatic adenocarcinoma	
Left axillary lesion	Positive: CK7 and AR positive Negative: PAX8, RCC, CK20, TTF1, ER, PR, HER2/neu, and PD-L1 MMR intact		Initial Dx: ulcerated invasive adenocarcinoma Final Dx: primary eccrine carcinoma	Somatic mutations: *PALB2* pathogenic variant, p.M723fs, exon 5, c.2167_2168delAT, VAF 49% *PALB2* pathogenic variant, p.Val504fs, exon 4, c. 1510dupG, VAF 29% *BRCA1* mutation of uncertain significance, p.Thr528Pro, exon 10, c.1582A>C, VAF 9% Germline mutations: Heterozygous for a pathologic variant in the *PALB2* gene with c.2167_2168del (p.Met723Vfs∗21) mutation. Notably, this germline mutation was initially found in his RCC, but without LOH.
Case 2				
Left forearm	Positive: CK5/6 and BerEP4, focal CEA Negative: CK20, chromogranin		Carcinoma with ductal differentiation; eccrine carcinoma either hidradenocarcinoma or porocarcinoma	
Left axillary lymph node	Positive: CK7, GATA3 Weak expression of PMS2 Negative: PAX8, TTF1, CK20, CDX2, NKX3.1, PDL-1, ER, PR MSI stable MMR intact Her2 amplification positive by FISH (HER2/CEP17 ratio 2.33, HER2 copy number 3.88)		Metastatic carcinoma	Left axillary lymph node: *BRCA1* somatic copy number loss with a pathogenic germline *BRCA1* p.Lys918fs chr17:41244796 mutation. *DDX3x* c.864+1G>C splice region variant loss of function (VAF 84.8%) *TP53* p.P89fs frameshift loss of function (VAF 79.0%) *CDKN1B* p.Leu41fs frame shift loss of function (VAF 42.8%) Blood germline testing: Heterozygous for a pathologic variant in the *BRCA1* gene c.2751del (p.Lys918Serf∗82) mutation Heterogenous for a pathologic variant *PMS2* c.2528 G>A (p.Cys843Y)

*AR*, Androgen receptor; *BRCA1*, breast cancer gene 1; *CDKN1B*, cyclin-dependent kinase inhibitor 1B; *CEA*, carcinoembryonic antigen; *CK*, cytokeratin; *ER*, estrogen receptor; *HER2*, human epidermal growth factor receptor 2; *IHC*, immunohistochemistry; *LOH*, loss of heterozygosity; *MMR*, mismatch repair; *PALB2*, partner and localizer of BRCA2; *PAX*, paired box gene; *PD-L1*, programmed death-ligand 1; *PMS2*, postmeiotic segregation increased 2; *PR*, progesterone receptor; *RCC*, renal cell carcinoma; *TP53*, tumor protein p53; *TTF1*, thyroid transcription factor-1; *VAF*, variant allele frequency.

### Case 2

An 88-year-old male presented with a red, eroded left forearm lesion, which was resected. Pathology revealed carcinoma with ductal differentiation positive for CK5/6 and BerEP4, with focal carcinoembryonic antigen positivity. CK20 and chromogranin were negative. A year later, he developed shortness of breath, prompting further evaluation. A computed tomography scan showed diffuse lymphadenopathy, increased pulmonary nodules, and a new left adrenal nodule. Positron emission tomography imaging confirmed extensive hypermetabolic metastatic disease involving multiple lymph nodes, bilateral pulmonary nodules, an adrenal nodule, and osseous lesions. A biopsy of the left axillary adenopathy revealed metastatic carcinoma with glandular/ductal differentiation resembling the initial skin biopsy. IHC showed CK7 and GATA binding protein 3 positivity but was negative for PAX8, TTF1, CK20, CDX2, and NKX3.1. Estrogen receptor and progesterone receptor were negative, and HER2 was equivocal (2+ by IHC), with HER2 amplification confirmed by FISH (HER2/CEP17 ratio 2.33, HER2 copy number 3.88). Histological similarities between the initial and metastatic biopsies suggested EC rather than breast carcinoma given the absence of breast lesions, strong CK5/6 expression (typically seen only in basal-like breast carcinomas), and an unusual metastatic pattern (Tables I and II). Genetic testing on the axillary lymph node was negative for programmed cell death protein ligand 1 and showed intact mismatch repair, though PMS2 expression was weak by IHC. Additionally, he had a *BRCA1* somatic copy number loss with a pathogenic germline *BRCA1* p.Lys918fs mutation. Hematologic germline testing revealed heterozygosity for the pathogenic *BRCA1* p.Lys918Serf∗82 variant and pathogenic *PMS2* p.Cys843Tyr variant, consistent with hereditary breast and ovarian cancer syndrome and Lynch syndromes, respectively (Table II).

**Fig 2:**
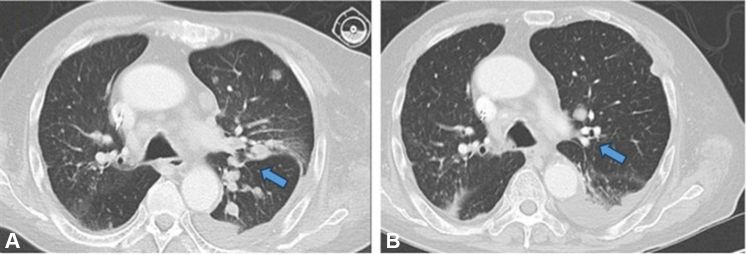
CT imaging of the chest for case 2 before (**A**) and after (**B**) receiving talazoparib. **A,** CT imaging of the chest showing numerous pulmonary metastatic foci (*blue arrow*) before receiving talazoparib. **B,** CT imaging of the chest 4 days after initiating talazoparib showed a decrease in the numerous metastatic foci (*blue arrow*). *CT*, Computed tomography.

After extensive multidisciplinary tumor board discussions and second opinions from other tertiary cancer centers, urgent treatment was recommended due to disease progression, postobstructive pneumonia, pathologic rib fractures, and suspected malignant pleural effusion. He was admitted with respiratory failure requiring intubation and started PARPi therapy with talazoparib (1 mg daily). After 4 days, computed tomography imaging showed a reduction in pulmonary metastatic foci (Fig 2) with significant clinical improvement allowing for extubation. Due to fatigue, talazoparib was dose-reduced to every other day with continued response.

## Discussion

ECs are rare cutaneous neoplasms of adnexal structures first described in 1865 and account for 0.005% to 0.01% of all malignant cutaneous neoplasms.^1^ While their etiology remains unclear, ECs are believed to originate from the acrosyringium *de novo* or malignant transformation of poromas.^1^ EC typically occurs in individuals aged 60 and older, most commonly found on the extremities.^1^ Risk factors include UV radiation, immunosuppression, and genetic mutations, most frequently tumor protein p53, retinoblastoma 1, and HRas proto-oncogene.^1^ Skin adnexal carcinomas are heterogeneous, sporadic, and infrequently sequenced. Some variants share histological and IHC features with glandular carcinomas from other organs, such as the breast (including Paget’s disease) and salivary glands—reflecting the evolutionary link between mammary glands and modified apocrine sweat glands.^6^

The molecular landscape of EC remains poorly understood, though some recurrent somatic genomic alterations have been identified.^7^ Oncogenic drivers of adnexal neoplasms include somatic alterations in intracellular signaling pathways such as mitogen-activated protein kinase and *PIK3CA*, HRas proto-oncogene, and epidermal growth factor receptor.^7^ Malignant progression has been linked to tumor suppressor loss, particularly in tumor protein p53, cyclin-dependent kinase inhibitor 2A, retinoblastoma 1, and adenomatous polyposis coli.^7^ In our first case, pathogenic variants were identified in *PALB2* (p.Met723fs, variant allele frequency [VAF] 49%; p.Val504fs, VAF 29%) and *PIK3CA* (p.His1047Arg, VAF 32%), alongside *BRCA1* VUS (VAF 9%). *PALB2* germline pathogenic variants are associated with increased risks for breast (53% in females, 1% in males), ovarian (5%), and pancreatic cancers (2% to 3%).^8^ The second case had *BRCA1* somatic copy number loss with a pathogenic germline *BRCA1* p.Lys918fs mutation. Additionally, he was heterozygous for a pathogenic *PMS2* p. Cys843Tyr variant, linked to Lynch syndrome, which increases colorectal cancer risk.^9^ While mutations in DNA damage repair (DDR) genes are well studied in breast and gastrointestinal cancers, their role in EC is not well characterized.^4^^,^^10^^,^^11^ Whole exome sequencing on 14 formalin-fixed paraffin-embedded ECs showed that the most common mutational signature was UV-induced (11/14 cases).^10^ Of these 14 ECs, 13/14 had alterations in homologous recombinational repair (HRR) genes. While this study did not assess the germline status of these HRR genes, the presence of HRR defects in the EC tumors suggests the HRR pathway's involvement in EC pathogenesis.

PALB2 is an integral component of the BRCA complex required for HRR and an important mediator in hereditary breast cancer pathogenesis.^11^ To date, *PALB2* mutations have not been linked to an increased risk of cutaneous neoplasms.^8^ In the first case, the patient harbored a *PALB2* p.Met723fs with a VAF near 50%, along with a second somatic frameshift *PALB2* variant, suggesting loss of heterozygosity of PALB2 function may have driven the malignant transformation of the EC. Conversely, *PALB2* is not associated with an increased rate of RCC, and molecular analysis of his RCC shows he still maintains a functional *PALB2* allele.^11^

While BRCA1 defects are linked with a highly penetrant cancer syndrome (involving breast, ovarian, and pancreatic cancer), their association with skin cancers remains unestablished, as ECs were excluded from prior studies.^12^ In these cases, the first patient carried a *BRCA1* p.Thr528Pro VUS, while the second had a *BRCA1* somatic copy number loss along with a pathogenic germline *BRCA1* p.Lys918fs mutation. Germline testing of the second case revealed heterozygosity for *BRCA1* p.Lys918Serf∗82, consistent with hereditary breast and ovarian cancer syndrome. Notably, this patient’s first cancer presentation occurred in the eighth decade of life as a cutaneous glandular carcinoma.

While most cutaneous adnexal carcinomas are sporadic, some are linked to inherited tumor syndromes including Muir-Torre syndrome, Cowden syndrome, and CYLD cutaneous syndrome.^13^ However, these syndromes typically present with sebaceous tumors, trichilemmomas, or cylindromas, rather than EC. To date, no genetic syndromes have been definitively associated with EC, and its inheritability remains unclear due to their rarity.^7^ However, the overrepresentation of HRR defects in a case series of 14 EC tumors suggests that individuals with germline HRR mutations may experience a greater incidence of EC. The absence of EC in known HRR-related cancer syndromes may be due to EC’s low incidence, even among genetically predisposed individuals.

This report identified EC in patients with cancer predisposition syndromes linked to DDR germline alterations associated with HRD. Given EC’s rarity, its genetic drivers remain poorly understood, and germline testing is not routinely performed in clinical management.

Our findings suggest a potential association between EC and cancer predisposition syndromes warranting further research into the role of DDR gene mutations in EC pathogenesis. This would have broad implications in both the workup and treatment of EC. This has implications for diagnosis, treatment, and genetic counseling. If validated, germline testing, cancer screening, and genetic counseling for at-risk relatives should be considered in EC patients. This is exemplified by our first case, in which a patient later developed pancreatic cancer, a malignancy associated with *PALB2* germline mutations. Currently, germline panels for *PALB2* and *BRCA1/2* are utilized for breast, ovarian, and pancreatic cancer. Expanding the use of these panels to include EC patients could improve risk assessment and inform treatment, potentially establishing a new standard of care.

There are no standardized treatments for EC beyond surgery, chemotherapy, and radiation.^1^^,^^14^ Mohs micrographic surgery and wide local excision are recommended for early-stage, resectable disease.^1^ While radiation therapy guidelines remain unclear, it is typically used adjuvantly for residual disease or with chemotherapy for unresectable tumors.^1^^,^^14^ Platinum-based chemotherapy has been employed, but ECs are largely resistant to cytotoxic agents, resulting in poor outcomes.^1^ Targeted therapies such as sunitinib, vismodegib, and HER2 inhibitors have been explored, as well as pembrolizumab for programmed cell death protein ligand 1 positive metastatic disease, though with limited success.^14^ Given the indication of germline HRD-associated mutations in these cases, PARPi may represent a novel treatment option. HRD has been linked to enhanced PARPi response in breast and ovarian cancers, where it serves as a predictive.^15^ Notably, germline BRCA1/2-mutated ovarian cancers or those with high HRD status exhibit longer progression-free survival with PARPi therapy.^15^ If the association between EC and HRD is validated, PARPi could provide a new avenue of therapy in these tumors.

## Conclusion

Sweat gland cancers can be the first presentation of a cancer predisposition syndrome. We have described sweat gland tumors with HRD-associated germline *PALB2*, *BRCA1*, and *PMS2* mutations. We, therefore, recommend screening EC patients for cancer predisposition genes. Additionally, our study highlights that there may be a role for PARPi in EC with actionable alterations, underscoring the importance of molecular phenotyping in these patients for precision oncology treatment.

## Conflicts of interest

None disclosed.
